# Access to health in city slum dwellers: The case of Sodom and Gomorrah in Accra, Ghana

**DOI:** 10.4102/phcfm.v8i1.822

**Published:** 2016-03-29

**Authors:** Frances E. Owusu-Ansah, Harry Tagbor, Mabel Afi Togbe

**Affiliations:** 1 Department of Behavioural Sciences, School of Medical Sciences, Kwame Nkrumah University of Science and Technology, Ghana; 2 Department of Community Health, School of Medical Sciences, Kwame Nkrumah University of Science and Technology, Ghana; 3 Military Hospital, Accra, Ghana

## Abstract

**Background:**

Rapid rural-urban migration of people to cities is a reality around the globe that has increased city slum dwellers. Sodom and Gomorrah is a city slum located in the heart of Accra, Ghana. Like other slums, it lacks basic amenities necessary for dwellers’ quality of life. This study describes residents’ access to health and factors associated with the use of healthcare facilities.

**Methods:**

Questionnaires were administered in systematically selected shacks across the entire slum. Data on demographic characteristics, existent health facilities and number of users, health-insured residents and knowledge of common diseases were collected.

**Results:**

Majority of the residents were from the northern parts of Ghana, relative to the south and a few of them come from other parts of West Africa. Seventy-one percent of residents had never visited a health facility in the last 5 years. When necessary, they access health care from drug stores (61.1%) or hospitals (33.1%). Residents’ age, educational status, income, health knowledge and membership of National Health Insurance Scheme were significantly (*p* < 0.05) associated with the use of healthcare facilities. Younger residents and those without National Health Insurance Scheme membership, formal education, no knowledge of common illnesses and regular income were significantly less likely to use a healthcare facility. For most residents, neither distance (73.2%) nor transportation to health facilities was a problem (74.1%).

**Conclusion:**

Conditions of profound environmental hazards, overcrowding, poor-quality housing and lack of health care in Sodom and Gomorrah pose grave threats to the health of the inhabitants. Multisectoral interventions and resource mobilisation championed by the Ministry of Local Government and Rural Development are needed to alter the trend.

## Background

A slum, according to the United Nations and UN-Habitat, is a run-down area of a city characterised by poor substandard structural quality of housing, insecure residential status, squalor, overcrowding and lack of basic amenities.^1,2,3^ Slums are usually inhabited by the very poor or socially disadvantaged groups, with a yearly increase in city slum dwellers in developing countries because of population growth and rural-urban migration.^1,2,3,4^ Poverty in the slums is reflected in the inadequate access to basic human needs such as clean water, nutrition, clothing, shelter, education and health care.^5,6^ Slum dwellers are caught up in a vicious cycle of economical and psychological poverty. Stack poverty makes it difficult for them to afford many essentials of life; they experience grave deprivation pushing them into a state of despondency.^1,2,3^

City slums may geographically be close to healthcare facilities, yet residents are deprived of access to needed health care resulting in negative economic and health consequences.^7^ The overcrowding and poor housing coupled with the dirt and squalor and lack of financial resources contribute to the presence and spread of varied infectious diseases in slums, affecting women and children mainly.^1,2,3,8,9,10^ Maternal mortality, high vulnerability to HIV infection, high unmet need for family planning and developmental challenges in children and adolescents are just a few of the many negative results of poor access to health in city slums.^10,11,12,13,14,15^

Improving living conditions and access to quality health care for all, particularly for slums dwellers, is central to development and speedy realization of the Millennium Development Goals (MDGs) in any country. Efforts towards achieving the first MDG goal, eradication of extreme poverty, through cross-cutting appears more urgent in slums where poverty is demonstrably present.^1,6,8^ Three of the MDGs − 4, 5, and 6 − specifically focus on health and seek to reduce infant and maternal mortality and combat diseases such as HIV and malaria, conditions which are unfortunately rampant in slums.^3,16^ To be effective, interventions aimed at making lasting changes must be context-specific, empowering and evidence-based. It is in this regard that research in this area is essential if strategies are to produce the desired outcomes.

The purpose of the study was to examine health issues in Sodom and Gomorrah in Accra, Ghana, and factors that may be associated with accessing healthcare facilities in the last year. This study is timely in the light of the fact that even though a plethora of studies on slums exist, very few have looked at the situation in sub-Saharan Africa and studies on Ghana are practically nonexistent. Because slums around the globe share similar characteristics of poverty and deprivations in many spheres of life, including health care,^3,4,8,10,11,14,16,17,18,19^ it is expected that findings from this study can provide insights into some of the health issues of other city slums within the country and sub-Saharan Africa for appropriate and targeted interventions.

## Methods

### Study area and population

The survey was conducted in Sodom and Gomorrah, a slum within Accra city, near the Korle Bu Teaching Hospital (KBTH). It stretches across 146 hectares and houses an estimated 25 000 to 40 000 residents. Residents are ethnically diverse, mostly poor, barely educated and generally unemployed or engaged in odd, nonpermanent jobs. Sodom and Gomorrah is characterised by poor housing, dirt and squalor, overcrowding and inadequate access to safe and clean water, sanitation and other infrastructure. It is one of the world’s digital dumping grounds, where millions of electronic waste products from the West are crudely processed each year.^20^ The study population consisted of adults in the Sodom and Gomorrah community who were heads of their respective dwellings or households.

### Study size and sampling technique

This was a cross-sectional study. We conceptualised that access to health care in the slum was low and influenced by factors such as availability of health facilities, pattern of health facility use, based in part on knowledge of common illnesses, and on acquisition of health insurance to facilitate use of available health services. Based on this, we selected a total of 465 adults in the community who were heads of their respective dwellings or households. This sample size was deemed sufficient to estimate with 95% confidence that the proportion of Sodom and Gomorrah dwellers who used a healthcare facility in the last 1 year prior to this survey will not differ from 55% (the proportion of rural populations in Ghana who consult medical personnel)^21^ by 5 percentage points and accounting for 10% nonresponse. For convenience and with the aim of covering all parts of the slum, the slum was divided into four areas and in each of these areas, the first household was selected and entered and subsequently every third household were entered till the required sample size was achieved.

### Data collection and analysis

A structured questionnaire with close-ended questions on respondent demographics, use of health facility in the last 1 year, membership of National Health Insurance Scheme (NHIS) and knowledge of causes, signs and symptoms, treatment and prevention of common illnesses in the community was used for data collection. Other data collected on potential barriers to the access and use of healthcare facilities included attitude of healthcare providers, distance to nearest health facility and access to transportation. The questionnaire was independently reviewed and edited by the investigators to ensure quality control and appropriateness for study. The questionnaire was pretested in Makola in the central business district of Accra amongst 50 head porters and truck pushers at the close of day when they were readily available. Pretesting of the questionnaire was done in this group because the investigators perceived that they are similar to the slum residents in demographic characteristics and hassled life styles. Pretesting of the questionnaire did not reveal any need for changes.

A research assistant and her team were trained on the administration of the questionnaire to ensure uniformity and consistency in their approach. The questionnaire was administered individually to participants by a team led by the research assistant moving from dwelling to dwelling within the slum over a 2-week period in December 2011.

Respondents were approached by the research assistant and her team who explained in detail the purpose of the study and solicited participation. Data were entered in Microsoft Office Excel, 2007. A composite indicator for health knowledge based on knowledge of causes, signs and symptoms, and prevention of malaria and cholera and common illnesses mentioned was constructed. Questions assessing knowledge were grouped under six blocks, which are causes of malaria, signs and symptoms of malaria, prevention of malaria, signs and symptoms of cholera, prevention of cholera and knowledge of other illnesses in the slum. Respondents’ knowledge was graded as good if they provided at least one correct response for each block. Respondents’ knowledge was graded as fair if they provided at least one correct response for only four or five blocks. Respondents’ knowledge was graded as poor if they provided at least one correct response for only three or less blocks. Stata 12.1^22^ was used for data processing and analysis. Chi-squared test was used to assess statistical differences between independent categorical variables in the levels of use of formal health care.

Risk ratio (RR) estimates and their confidence intervals were estimated by using the modified Poisson regression with a robust error variance. Adjusting the RR for other predictors or potential confounders was done by adding them to the model statement. A *p*-value of ≤ 0.05 is considered statistically significant.

## Ethics statement

Ethical approval for this study was granted by the Committee on Human Research, Publications and Ethics, Kwame Nkrumah University of Science and Technology, School of Medical Sciences, Kumasi, Ghana. Written informed consent was sought from all participants and consent obtained before questionnaires were administered. Illiterate participants thumb printed and/or gave verbal consent after they had been provided with adequate information about the study and had all their concerns and/or questions answered.

## Results

We sampled a total of 465 slum dwellers. Two were excluded from the analysis because of nonresponses. The Sodom and Gomorrah community has no hospitals or clinics. The residents said they mostly accessed health care from two drug stores in the slum and from herbalists. A few attended the Cathedral Clinic nearby in the past and only one respondent had ever attended the KBTH, which is a tertiary health facility in the city of Accra. Some demographic characteristics of the participants are presented in Table 1. The majority of respondents were male residents (64.4%). The median age of respondents was 24 years and ranged from 18 to 59 years. The vast majority of respondents (70.5%) were engaged in nonformal employment, 15.9% had formal employment and the rest (13.5%) were unemployed. Around 60% of the respondents had had at least a primary-level of education. Majority of the residents (71.2%) had never visited a health facility anytime in the past. The few (28.8%) who claimed they did could not recollect their last visit but admitted doing so sporadically in the last 1 or more years ago. When specifically asked, about 29% of them said they had accessed a formal healthcare unit (hospital, clinic or health centre) in the past 1 year. To meet their health needs, 61.1% of the residents relied on drug stores, self-medication (3.3%) or consulted the herbalist (2.4%).

**TABLE 1:** Some characteristics of residents of Sodom and Gomorrah.

Characteristics	*n*	%
**Sex**		
Male	298	64.4
Female	165	35.6
**Age**		
18–21	126	30.66
22–25	144	35.04
26 and above	141	34.31
Mean (SD)	25.6	(7.5)
Median (interquartile range)	24	(7)
**Ethnic origin**		
Southern	164	35.9
Northern	261	57.1
Foreign	32	7.0
**Ever visited a health facility**		
During last year	72	15.9
More than a year ago	58	12.8
Never	322	71.2
**Use of health facility**		
Yes	136	29.4
No	327	70.6
**NHIS membership**		
Yes	90	19.4
No	373	80.6
**Meeting health needs**		
Hospital	149	33.1
Chemical shop	275	61.1
Herbalist	11	2.4
Self-medication	15	3.3
**Transportation to health facility**		
No problem	338	74.1
A problem	118	25.9
**Distance to health facility**		
No problem	333	73.2
A problem	122	26.8

NHIS, National Health Insurance Scheme; SD, standard deviation.

Majority of them (80.6%) were not registered members of the NHIS at the time of the survey. They cited lack of money (45.5%) for the initial registration as the main reason for not having a national health insurance membership. Other reasons included inconvenience (13.4%), no need for it (9.1%) and other unexplained reasons (14.7%). The respondents were predominantly from the northern parts of Ghana, relative to the south. A few of them were from the West African subregion.

According to the residents, malaria and diarrhoeal diseases were the most prevalent illnesses in the slum because of the prevailing poor sanitation conditions. They also mentioned mental illness, HIV and convulsion in the young, though these were less frequent. Using malaria and cholera, we gauged residents’ health awareness by asking about the causes, symptoms and signs, and methods of prevention of the two diseases. Majority of the respondents (47.7%) had good knowledge about the causes, symptoms and signs of these illnesses and measures to prevent their occurrence (Table 2).

**TABLE 2:** Assessment of residents’ knowledge on common illnesses.

Characteristics	*n*	%
**Prevalent illnesses**		
Malaria	153	34.5
Diarrhoea	86	19.4
Respiratory tract infections	47	10.6
HIV	14	3.2
Mental illness	53	12.0
Other	90	20.3
**Causes of malaria**		
Mosquito bites	304	67.7
Choked gutters	27	6.0
Rubbish damps	23	5.1
Open gutters	8	1.8
Stagnant waters	6	1.3
Others	81	18.0
**Malaria prevention**		
Clean environment	176	40.7
Insecticide treated net use	126	29.2
Screen entrances	15	3.5
Use insecticides	71	16.4
Others	44	10.2
**Malaria symptoms**		
Fever	149	33.1
Headache	52	11.6
Chills	62	13.8
Vomiting	35	7.8
Weakness	66	14.7
Loss of appetite	16	3.6
Others	70	15.6
**Symptoms of cholera**		
Watery stools	293	64.8
Vomiting	55	12.2
Weakness	30	6.6
Loss of appetite	6	1.3
Others	68	15.0
**Cholera prevention**		
Good hygiene	251	63.5
Eat clean food	72	18.2
Use clean water	4	1.0
Clean hands	57	14.4
Hygienic food handling	11	2.8
**Overall knowledge score**		
Poor	55	11.88
Fair	187	40.39
Good	221	47.73

Factors associated with the use of formal healthcare facilities amongst the slum dwellers are presented in Tables 3 and 4. Residents aged 26 years or more and those having primary education (6 years of basic education) or higher were significantly more likely to use a formal healthcare facility. However, residents with poor knowledge of causes, signs and symptoms, and prevention of common illnesses in the community and those without jobs or regular incomes and those without NHIS membership were significantly less likely to use a formal healthcare facility. Ethnic origin and gender seemed not to have a significant effect on the use of formal healthcare facilities. Adjusting for the estimated RRs by including all these predictors in the model showed that the level of education, membership of NHIS, overall knowledge of common illnesses in the community and income level are the main factors that determined whether a resident used a formal health. Neither distance (73.2%) nor transportation to health facility was a problem (74.1%) hindering their use of formal health care by residents.

**TABLE 3:** Factors associated with utilisation of health care by residents of Sodom and Gomorrah.

Associated factors	Used healthcare facility						*p*-value

	No		Yes		Total		
		
	*n*	%	*n*	%	*n*	%	
**Sex**							
Male	204	62.39	94	69.12	298	64.36	0.168
Female	123	37.61	42	30.88	165	35.64	
**Age category**							
18–21	94	32.64	32	26.02	126	30.66	0.015
22–25	108	37.5	36	29.27	144	35.04	
26 and above	86	29.86	55	44.72	141	34.31	
**Marital status**							
Married	117	36.34	55	41.67	172	37.89	0.288
Not married	205	63.66	77	58.33	282	62.11	
**Education**							
No education	146	45.48	35	26.52	181	39.96	< 0.001
Primary education	121	37.69	61	46.21	182	40.18	
Secondary education	54	16.82	36	27.27	90	19.87	
**Ethnic origin**							
Southern	109	33.75	55	41.04	164	35.89	0.003
Northern	198	61.3	63	47.01	261	57.11	
Foreign	16	4.95	16	11.94	32	7	
**Occupation**							
Earn regular income	42	12.96	31	23.13	73	15.94	0.007
No regular job/income	282	87.04	103	76.87	385	84.06	
**NHIS membership**							
Yes	48	14.68	42	30.88	90	19.44	< 0.001
No	279	85.32	94	69.12	373	80.56	
**Knowledge of symptoms**							
Some knowledge	46	14.07	30	22.06	76	16.41	0.034
No knowledge	281	85.93	106	77.94	387	83.59	
**Overall knowledge score**							
Poor	30	9.17	25	18.38	55	11.88	0.006
Fair	129	39.45	58	42.65	187	40.39	
Good	168	51.38	53	38.97	221	47.73	
**Has chronic illness**							
No	303	94.39	123	90.44	426	93.22	0.125
Yes	18	5.61	13	9.56	31	6.78	
**Transportation to facility**							
Not a problem	254	79.38	84	61.76	338	74.12	< 0.001
A problem	66	20.63	52	38.24	118	25.88	
**Distance to facility**							
Not a problem	241	75.31	92	68.15	333	73.19	0.115
A problem	79	24.69	43	31.85	122	26.81	
**Staff attitude**							
Not a problem	177	55.14	88	65.19	265	58.11	0.047
A problem	144	44.86	47	34.81	191	41.89	

NHIS, National Health Insurance Scheme.

**TABLE 4:** Estimates of relative risk of predictors of utilisation of health care by residents of Sodom and Gomorrah.

Predictors of healthcare use	Risk ratio	95% Confidence interval	*p*-value	IRR	95% Confidence interval	*p*-value
**Sex**						
Male	Reference			Reference		
Female	0.81	0.59–1.10	0.176	1.14	0.82–1.57	0.434
**Age category**						
18-	Reference			Reference		
22-	0.98	0.65–1.48	0.94	1.08	0.69–1.66	0.732
26-	1.54	1.06–2.21	0.021	1.51	0.99–2.28	0.053
**Marital Status**						
Married	Reference			Reference		
Not married	0.85	0.63–1.14	0.285	1.34	0.96–1.87	0.082
**Education**						
No education	Reference			Reference		
Primary education	1.73	1.20–2.48	0.003	1.57	1.06–2.30	0.023
Secondary education	2.07	1.39–3.05	< 0.001	1.72	1.11–2.66	0.014
**Ethnic origin**						
Southern	Reference			Reference		
Northern	0.72	0.53–0.97	0.034	0.98	0.71–1.34	0.912
Foreign	1.49	0.99–2.24	0.055	1.18	0.69–2.01	0.541
**Occupation**						
Earn regular income	Reference			Reference		
No regular job/income	0.63	0.46–0.86	0.004	0.70	0.50–0.96	0.031
**NHIS membership**						
Yes	Reference			Reference		
No	0.54	0.40–0.71	< 0.001	0.55	0.39–0.75	< 0.001
**Knowledge of symptoms**						
Some knowledge	Reference			Reference		
No knowledge	0.69	0.50–0.95	0.026	0.61	0.43–0.85	0.004
**Overall knowledge score**						
Poor	Reference			Reference		
Fair	0.68	0.47–0.97	0.038	0.64	0.42–0.96	0.033
Good	0.53	0.36–0.76	0.001	0.44	0.28–0.66	< 0.001
**Has chronic illness**						
No	Reference			Reference		
Yes	1.45	0.93–2.25	0.097	1.45	0.86–2.44	0.158
**Transportation to facility**						
Not a problem	Reference			Reference		
A problem	1.77	1.34–2.33	< 0.001	1.62	1.17–2.23	0.003
**Distance to facility**						
Not a problem	Reference			Reference		
A problem	1.28	0.94–1.71	0.108	1.01	0.71–1.43	0.943
**Staff attitude**						
Not a problem	Reference			Reference		
A problem	0.74	0.54–1.00	0.051	0.77	0.55–1.07	0.122

NHIS, National Health Insurance Scheme.

## Discussion

In this study, we assessed utilisation of formal healthcare services by residents of Sodom and Gomorrah as a measure of access to and use of health care. We found that the majority of those sampled were youthful but unskilled, often jobless, and paid less attention to their health and general welfare. Majority of respondents had at least a primary-level education and a good overall knowledge about prevalent illnesses amongst residents of the slum. Just about 2 out of 10 respondents had an NHIS membership. A resident with NHIS membership was more than twice as likely to use a formal healthcare facility. Sodom and Gomorrah is centrally located in Accra and just about 300 m from the KBTH and even closer to other smaller health facilities. Indeed, the respondents indicated that neither distance nor access to transportation hindered them from using a health facility. Despite the central location of the slum and its proximity to healthcare facilities, it was interesting to note that less than 3 out of 10 used formal health care when they had need for it. They chose instead to seek from other sources. However, Agarwal and colleagues^16^ suggest that this is not always the case. They argued that if health authorities appropriately respond, proximity to formal healthcare facilities leads to improved health awareness, which positively influences healthcare use and health-seeking behaviour.^16^

The main health problems prevalent in this slum were malaria and diarrhoeal diseases. This is not surprising as the community has no pipe-borne water or good sanitary facilities. Sanitation is generally poor. There are no well-constructed gutters or drainage systems to allow easy flow of water. The few open gutters are choked with debris, resulting in dirty stagnant ponds and flooding during the rainy season. Houses are overcrowded and all-purpose. This is typical of slums as was observed in other studies conducted in slums.^5,6,8,23^ Living in slums under deprived conditions is a major cause of ill-health and slum dwellers suffer disproportionately from ill-health throughout their life course.^3,5,24,25,26,27^

It was observed that residents of Sodom and Gomorrah live in poor shacks primarily built or held together with old roofing sheets, plywood and/or cardboard papers. Children appeared neglected and malnourished, whilst adults loiter around smoking marijuana, sometimes with young children looking on. These conditions characterise Sodom and Gomorrah as a slum as defined operationally by the UN.^28^ However, it is difficult to explain why residents ‘feel’ at home under such profound deprivation prevailing in Sodom and Gomorrah. It is also needless to argue the circumstances leading to the creation of this slum as the demographic and socio-economic indicators are similar to those found in slums worldwide.^1,2^ However, the existence of such slums may be an expression of social exclusion. Slums appear to have intergenerational negative effects because children born in the slum have a slim chance of breaking through the poverty cycle. The significance of this study is its novelty and the information gleaned about the lives of slum dwellers within the city of Accra thus providing clues for effective interventions to improve the quality of their lives.

These results suggest that proximity of Sodom and Gomorrah to a tertiary health facility and many others in the centre of Accra did not necessarily encourage their patronage amongst the residents. However, just as it is the case for most Ghanaians, health-seeking behaviour is influenced by cultural and religious beliefs and practices.^29,30,31,32^ This study, however, did not include a qualitative component to help understand the complex social phenomenon of the health-seeking behaviours of the slum residents. Inclusion of such data could have informed findings and targeted interventions. Future studies need to include these psycho-social measures for greater understanding of the health-seeking behaviours of slum dwellers.

## Conclusion

Increasing urbanisation is a major factor in the creation and continued existence of the Sodom and Gomorrah slum. The slum reveals a ‘theatre of social exclusion’ where prevailing poor economic, educational and sociocultural conditions conspire as if to perpetuate each other’s reign. Conditions of profound environmental hazards, overcrowding and poor-quality housing and lack of health care or other essential services in Sodom and Gomorrah pose grave threats to the health of the inhabitants and probably to the rest of the city of Accra. Interventions requiring significant multisectoral effort and resource mobilisation championed by the Ministry of Local Government and Rural Development are needed to attempt a resolution of this crisis.
